# Supplementary material for: Challenges in Quantifying 8-OHdG and 8-Isoprostane in Exhaled Breath Condensate
Source: Antioxidants (Basel). 2022 Apr 25;11(5):830. doi: 10.3390/antiox11050830 (PMC9138069; doi:10.3390/antiox11050830)
Supplement: Supplementary file 1 [file antioxidants-11-00830-s001.zip › antioxidants-1658246-supplementary.pdf]

antioxidants

Supplementary Information

Challenges in quantifying 8-OHdG and 8-Isoprostane in exhaled breath condensate

Maud Hemmendinger[1*], Jean-Jacques Sauvain[1], Nancy B. Hopf[1], Guillaume Suárez[1] and Irina Guseva Canu[1]

1 Department of Occupational and Environmental Health, Center for Primary Care and Public Health (Unisanté), 1006, Epalinges-Lausanne, Switzerland; jean-jacques.sauvain@unisante.ch (J.J.S.); nancy.hopf@unisante.ch (N.H.); guillaume.suarez@unisante.ch (G.S); irina.guseva-canu@unisante.ch (I.G.C.)
* Correspondence: maud.hemmendinger@unisante.ch; Tel.: (+41 21 314 74 59);

Table S1. Description of the standard preparation with the corresponding concentrations

Label	volume of working solution at 500 pg/ml [µL]	Volume of H_2O [µl]	Volume of working solution of IS 2,5ng/ml [µL]	Volume of BHT [µL]	Total volume [µl]	Final concentration Std [pg/ml]	Final concentration IS [ng/ml]	BHT (mg/ml)
**Std 0**	0	800	200	10	1010	0	0,495	0,099
**Std 15**	30	770	200	10	1010	15	0,495	0,099
**Std 50**	100	700	200	10	1010	50	0,495	0,099
**Std 100**	200	600	200	10	1010	99	0,495	0,099
**Std 300**	600	200	200	10	1010	297	0.495	0.099
Before concentration								
**H20 0**	0	985	15	10	1010	0	0,037	0,099
**H20 5**	10	975	15	10	1010	5	0,037	0,099
**H20 15**	30	955	15	10	1010	15	0,037	0,099
**H20 25**	50	935	15	10	1010	25	0,037	0,099
**H20 50**	100	885	15	10	1010	50	0,037	0,099
						After concentration and reconstitution in 75µl		
						**0**	**0,495**	**1,320**
						**67**	**0,495**	**1,320**
						**200**	**0,495**	**1,320**
						**333**	**0,495**	**1,320**
						**666**	**0.495**	**1.320**

Std = not evaporated standards; H20 = standards that will be evaporated.

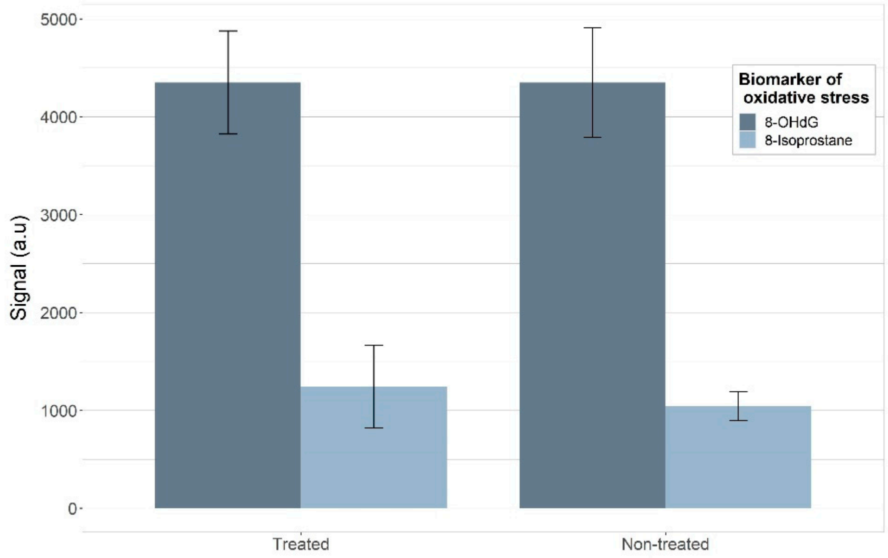

Figure S1. Effect of Tween-20 treatment on material surface on the signal (not corrected with IS) of 8-OHdg and 8- isoprostane. Error bars correspond to a minimum of nine independent repetitions.

Table S2. Main figure of merit of the analytical method for 8-OHdG in EBC

Method characteristics - 8-OHdG	Expected performances	Observed values	Conclusion
Calibration			
Function			linear
Concentration range [pg/ml]	0-1333		
Bias for std. 67 pg/ml	>15%	6,9%	Verified
Bias for std. 200 pg/ml	10%	4,9%	Verified
Bias for std. 333 pg/ml	10%	1,3%	Verified
Bias for std. 666 pg/ml	10%	1,0%	Verified
Bias for std. 1333 pg/ml	10%	0,4%	Verified
LOQ (a-priori estimate)	22 pg/ml (ech conc speedvac)		
Maximum accepted range	±13.2 pg/ml =60%*22 pg/ml		
Upper accepted value [pg/ml]	35,2	27,1	Verified
Lower accepted value [pg/ml]	8,8	23,5	Verified
LOD (1/3 LOQ) [pg/ml]		7,30	Verified
LOD in EBC [pg/ml]		0,5	Verified
Recovery			
Value EBC non spiked [pg/ml]	0,00		
EBC spiked 1 [pg/ml]	5,00	4,8	
EBC spiked 2 [pg/ml]	15,00	14,0	
Recovery spike 1 [%]	[90 - 110]	98±10	Verified
Recovery spike 2 [%]	[90 - 110]	89±8	Verified
Repetability (CV)			
EBC non spiked	< LOD	n.d.	
EBC spiked 1 (5 pg/ml)	20%	19,8	Verified
EBC spiked 2 (15 pg/ml)	10%	1,0	Verified
Accuracy			
Reference value EBC spiked 1 [pg/ml]	5,00		
u_{ref}	2,0		
Maximum accepted difference	25%		
Normalized range	2	0,14	Verified
Maximum accepted range [pg/ml]	6,25	6,02	Verified
Minimum accepted range [pg/ml]	3,75	4,53	Verified
Reference value EBC spiked 2 [pg/ml]	15,00		
u_{ref}	2,0		
Maximum accepted difference	10%		
Normalized range	2	-0,52	Verified
Maximum accepted range [pg/ml]	16,50	14,82	Verified
Minimum accepted range [pg/ml]	13,50	13,10	Not verified

Table S3. Main figure of merit of the analytical method for 8-isoprostane in EBC

Method characteristics - 8-isoprostane	Expected performances	Observed values	Conclusion
Calibration			
Function			linear
Concentration range [pg/ml]	0-1333		
Bias for std. 67 pg/ml	>15%	10,0%	Verified
Bias for std. 200 pg/ml	10%	-5,9%	Verified
Bias for std. 333 pg/ml	10%	4,5%	Verified
Bias for std. 666 pg/ml	10%	-2,6%	Verified
Bias for std. 1333 pg/ml	10%	1,0%	Verified
LOQ (a-priori estimate)	40 pg/ml (ech conc speedvac)		
Maximum accepted range	±24 pg/ml =60%*40 pg/ml		
Upper accepted value [pg/ml]	63,5	27,9	Verified
Lower accepted value [pg/ml]	15,9	18,3	Verified
LOD (1/3 LOQ) [pg/ml]		13,2	Verified
LOD in EBC [pg/ml]		1,0	Verified
Recovery			
Value EBC non spiked [pg/ml]	0,00		
EBC spiked 1 [pg/ml]	5,00	5,4	
EBC spiked 2 [pg/ml]	15,00	15,1	
Recovery spike 1 [%]	[90 - 110]	104±7	Verified
Recovery spike 2 [%]	[90 - 110]	95±6	Verified
Repetability (CV)			
EBC non spiked	< LOD	n.d.	
EBC spiked 1 (5 pg/ml)	20%	11,1	Verified
EBC spiked 2 (15 pg/ml)	10%	5,9	Verified
Accuracy			
Reference value EBC spiked 1 [pg/ml]	5,00		
u_{ref}	2,0		
Maximum accepted difference	50%		
Normalized range	2	0,38	Verified
Maximum accepted range [pg/ml]	7,50	7,34	Verified
Minimum accepted range [pg/ml]	2,50	4,23	Verified
Reference value EBC spiked 2 [pg/ml]	15,00		
u_{ref}	2,0		
Maximum accepted difference	15%		
Normalized range	2	0,04	Verified
Maximum accepted range [pg/ml]	17,25	16,87	Verified
Minimum accepted range [pg/ml]	12,75	13,29	Verified

Table S4. Results obtained for the calibration, QC and EBC samples

	**Calibration Curve**				**QC 5 (pg/ml)**		**Average EBC Concentration**
**8-OHdG**	Slope	Intercept	R^2	LOD (pg/ml)	Theoretical	Experimental	
Healthy (n=10)							<LOD
Asthma (n=7)	0.032	-0.0019	0.999	0.5	5	4.86 ±0.11 (n=4)	<LOD
COPD (n=9)							<LOD
**8-isoprostane**							
Healthy (n=10)	0.065	0.034	0.996	1.0	5	5.06±1.06 (n=4)	<LOD
Asthma (n=7)							<LOD
COPD (n=9)							<LOD

n = the number of measures.
